# Dietary patterns and colorectal cancer risk in a Korean population: A case-control study

**DOI:** 10.1097/MD.0000000000003759

**Published:** 2016-06-24

**Authors:** Yoon Park, Jeonghee Lee, Jae Hwan Oh, Aesun Shin, Jeongseon Kim

**Affiliations:** a Department of Cancer Control and Policy, Graduate School of Cancer Science and Policy; b Molecular Epidemiology Branch, Division of Cancer Epidemiology and Prevention, Research Institute, National Cancer Center; c Center for Colorectal Cancer, National Cancer Center Hospital, National Cancer Center, Goyang-si, Gyeonggi-do; d Department of Preventive Medicine, Seoul National University College of Medicine; e Cancer Research Institute, Seoul National University, Seoul, South Korea.

**Keywords:** case-control studies, colorectal cancer, dietary patterns, factor analysis, Korea

## Abstract

Colorectal cancer (CRC) has been recognized as one of the major malignancies in Korea. Analyses of dietary patterns can provide insight into the complex interactions of foods, nutrients, and biologically active components within a diet, which vary among populations. We aimed to investigate the associations between dietary patterns and colorectal cancer risk in Koreans. In a study of 923 cases and 1846 controls, principal component analysis was used to identify dietary patterns based on 33 predefined food groups using a 106-item semiquantitative food frequency questionnaire (SQFFQ). The associations between dietary patterns and CRC risk were assessed using binary and polytomous logistic regression models to estimate odds ratios (ORs) and 95% confidence intervals (CIs). Three dietary patterns (traditional, Westernized, and prudent) were derived. The proportion of total variation explained by 3 patterns was 24.2% for men and 25.3% for women. The traditional and prudent patterns were inversely associated with CRC risk [OR and 95% CI for the highest intake tertile of pattern score vs. the lowest = 0.35 (0.27–0.46) and 0.37 (0.28–0.48), respectively], whereas the Westernized pattern showed a positive association, especially among women [OR = 2.13 (1.35–3.34) for the highest tertile vs. the lowest]. A decrease in CRC risk among those with the highest intake of the prudent pattern was observed in all anatomical subsites in both men [OR = 0.36 (0.19–0.68) for proximal colon; 0.21 (0.12–0.36) for distal colon; 0.28 (0.18–0.44) for rectum] and women [OR = 0.28 (0.11–0.71); 0.27 (0.13–0.54); 0.45 (0.25–0.83)]. Our results indicate that individuals who prefer the Westernized dietary pattern should be made aware of their increased CRC risk. The traditional dietary pattern and the prudent pattern, which are rich in fruits and dairy products, are recommended for the Korean population to prevent CRC.

## Abstract 2

Supplemental Digital Content is available in the text

## 1 Introduction

Colorectal cancer (CRC) ranks globally as the third most commonly diagnosed cancer.^[1]^ Moreover, the rates of CRC have increased in economically developing countries, while in developed Western countries, the incidence and mortality rates of CRC have stabilized or decreased in recent years.^[1–3]^ The GLOBOCAN estimates presented for 2012 reported a high incidence of CRC,^[3]^ which is the third most common cancer in Korea. According to the Korean Central Cancer Registry, the age-adjusted incidences of CRC were 51.4 per 100000 for men and 28.0 per 100000 for women in 2012.^[4,5]^ The annual percent changes in CRC incidence were 5.6% in men and 4.3% in women between 1999 and 2012.^[5]^

This increase in CRC is thought to be associated with environmental factors such as changes in lifestyle due to Westernization and economic development in recent decades.^[6]^ Among diverse factors, diet has been regarded as a crucial factor that might modify the risk of CRC. Previous studies have demonstrated that different foods and their active constituents regulate epigenetic mechanisms that affect the colorectal carcinogenesis process.^[7,8]^ In addition, nutritional exposure during adolescence may result in persistent epigenetic changes that later influence CRC development.^[9]^ Previous meta-analyses of prospective studies have investigated the associations between CRC risk and intake of food groups, micronutrients, and macronutrients.^[10–16]^ The carcinogenicity levels of red and processed meat were recently reclassified by the World Health Organization as “probably carcinogenic to humans (Group 2A)” and “carcinogenic to humans (Group 1),” respectively, on the basis of sufficient evidence for CRC.^[17,18]^ However, in these analyses, which were conducted with individual dietary components, it was difficult to determine the relationships between health and a person's total diet, which includes a combination of various foods and nutrients.^[19,20]^ In this context, the multivariate data analysis of dietary patterns has emerged as a methodological approach to capture overall diet rather than a single food or nutrient and to assess the complex dietary exposures that are likely to be interactive or synergistic.^[16,21–23]^

Previous studies have reported inconsistent results of potential dietary risk for CRC in different populations with different cultures and backgrounds, especially those with varied diets and dietary patterns.^[19,24]^ In Korea, studies focusing on dietary patterns have been conducted since the early 2000s, especially with regard to cancer and the risk association.^[25,26]^ Furthermore, a previous study conducted in Korea suggested that risk factors might differentially influence cancer risk at different subsites.^[27]^ To date, there is little published information on the association between dietary patterns and CRC risk according to anatomical subsites in the Korean population. Therefore, the objective of the present study was to identify major dietary patterns among Koreans and to evaluate the associations of these patterns with CRC risk by gender, taking into account different anatomical subsites.

## 2 Methods

### 2.1 Study participants

**Figure 1:**
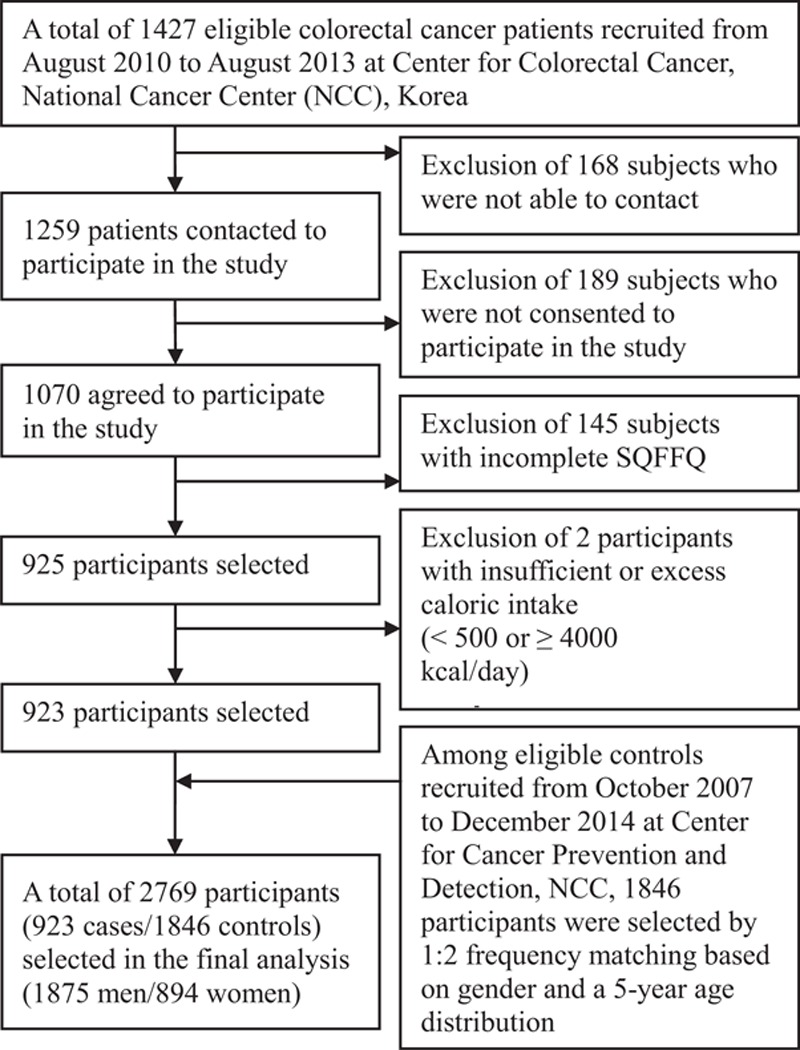
Flowchart of selecting study subjects in the study. SQFFQ = semiquantitative food frequency questionnaire.

To conduct a case-control study, newly diagnosed CRC patients were considered eligible for enrollment when they were admitted to the Center for Colorectal Cancer, National Cancer Center (NCC) in Korea from August 2010 to August 2013. We contacted 1259 of 1427 patients who underwent surgery for CRC, and 1070 patients agreed to participate in the study. A total of 923 patients were selected after the exclusion of 145 participants with incomplete semiquantitative food frequency questionnaires (SQFFQs), and 2 participants due to implausible energy intake (<500 or ≥4000 kcal/day). The selected cases were confirmed based on both pathology reports and chart review. We selected controls from among the visitors who underwent a health screening examination (a benefit program of the National Health Insurance) at the Center for Cancer Prevention and Detection, NCC in Korea, between October 2007 and December 2014. Among the visitors to the cancer-screening center, 14201 subjects agreed to participate in the study. A total of 9037 subjects remained after the exclusion of 5044 subjects with incomplete SQFFQs and 120 with implausible energy intakes. The data of the remaining subjects were linked with the Korea Central Cancer Registry and NCC medical charts to confirm that these subjects had not been diagnosed with CRC. Among the remaining subjects, 1846 controls were selected by a 1:2 frequency-matching to 923 cases by gender and age at 5-year intervals (Fig. 1).

### 2.2 Data collection

An interviewer collected information on subject's lifestyle and dietary intake by using a SQFFQ for CRC cases. Eligible controls were asked to complete a self-administered lifestyle questionnaire and a SQFFQ. All participants provided written informed consent and the study protocol was approved by the Institutional Review Board of the National Cancer Center (IRB Nos. NCCNCS-10-350 and NCC2015-0202). The lifestyle questionnaire included information on demographics, medical history, alcohol consumption, smoking habits, and physical activity. A validated SQFFQ was applied to determine dietary intake of the participants by collecting data on average intake frequency and portion size for 106 informative food items, which consisted of 410 different food compositions.^[28,29]^ The 106 food items listed in the SQFFQ were categorized into 33 food groups based on nutrient profiles and culinary usage (Appendix). For the specific analyses by cancer site, anatomical locations were abstracted from medical records and classified into 3 distinct locations: proximal colon (cecum, ascending colon, hepatic flexure, transverse colon, and splenic flexure), distal colon (descending colon, sigmoid-descending colon junction, and sigmoid colon), and rectum.

### 2.3 Assessment of dietary patterns

To define dietary patterns, an exploratory or a posteriori approach in which dietary patterns were derived empirically by applying statistical techniques to existing dietary data was conducted using a principal component analysis (PCA; PROC FACTOR).^[16,30]^ Extraction of principal components was followed by a varimax rotation (orthogonal) to achieve a structure with independent factors and greater potential for interpretability. The minimum eigenvalues of 1.0, the scree plot, and the interpretability of the factors were taken into account to determine which factors to retain with regard to dietary patterns. For each pattern, a factor score was calculated as a linear composite of the food groups with meaningful loadings (≥ |0.20|) for only that pattern.^[31,32]^ Gender-specific pattern scores were obtained by conducting separate factor analyses to identify major dietary patterns in men and women.

For further analysis, measurements of the dietary consumption of each pattern were adjusted for total energy intake using the linear residual regression method.^[33]^ The intake levels of each pattern were categorized into tertiles based on the distribution of the control groups. To understand the characteristics of the identified dietary patterns, analyses of nutritional intake were performed using CAN-Pro version 4.0 (Computer-Aided Nutritional Analysis Program, the Korean Nutrition Society, Seoul, Korea).

### 2.4 Statistical analyses

To compare the general characteristics of the cases and controls, Student *t* tests and χ^2^ tests were performed to compare continuous and categorical variables, respectively. Odds ratios (ORs) and 95% confidence intervals (CIs) were calculated across the tertiles of dietary patterns by logistic regression models after controlling for confounding factors, which included the potential risk factors of CRC. Multivariate models were adjusted for body mass index (BMI; defined by the criteria for the Asia-Pacific region),^[34]^ smoking status, alcohol consumption, physical activity, a first-degree family history of CRC, education level, occupation, marital status, monthly income, and total energy intake. Subgroup analyses of dietary patterns and CRC risk by cancer subsites (anatomical locations) were conducted using polytomous logistic regression methods across tertiles. The median intake of each tertile category of dietary pattern score was used as a continuous variable to test for trends. All statistical analyses were performed using SAS version 9.3 (SAS Institute Inc, Cary, NC). All statistical tests were 2-sided, and *P* values less than 0.05 were considered statistically significant.

## 3 Results

The socio-demographics and lifestyle characteristics of the 923 cases and 1846 controls are shown in Table 1. Due to the frequency-matched design, the age and gender distributions were similar between cases and controls. The mean age of the study population was approximately 56 years both in the cases (56.6 ± 9.7) and in the controls (56.1 ± 9.1). The mean of BMI was higher in the controls (24.1 ± 2.7) than in the cases (23.7 ± 3.3), which rejected the hypothesis that CRC patients might have higher BMI due to obesity. The proportion of obesity (BMI ≥ 25 kg/m^2^) was 33.9% in the controls and 30.9% in the cases, including 2.7% of controls and 3.6% of cases with BMI above 30 (*P* <0.001). The cases had lower marital status (*P* <0.001), higher unemployment rate including housekeeping (*P* <0.001), lower income (*P* <0.001), higher proportion of first-degree family history of CRC (*P* <0.001), and were less physically active (*P* <0.001) and less educated (*P* <0.001) compared with controls. The proportion of nonsmoker was similar in controls and cases (44.3% for both) and that of current smoker was higher in the cases (21.2%) than in the controls (18.5%) (*P* = 0.16). Considering status of alcohol consumption, the proportion of nondrinker was similar in controls (30.3%) and in cases (30.2%), and that of the former drinker was higher in the cases (14.0%) than in the controls (9.2%), whereas that of the current drinker was higher in the controls (60.5%) than in the cases (55.8%) (*P* <0.001). In the subsequent analyses, these variables were considered potential confounding factors in the association with the risk of CRC.

**Table 1:**
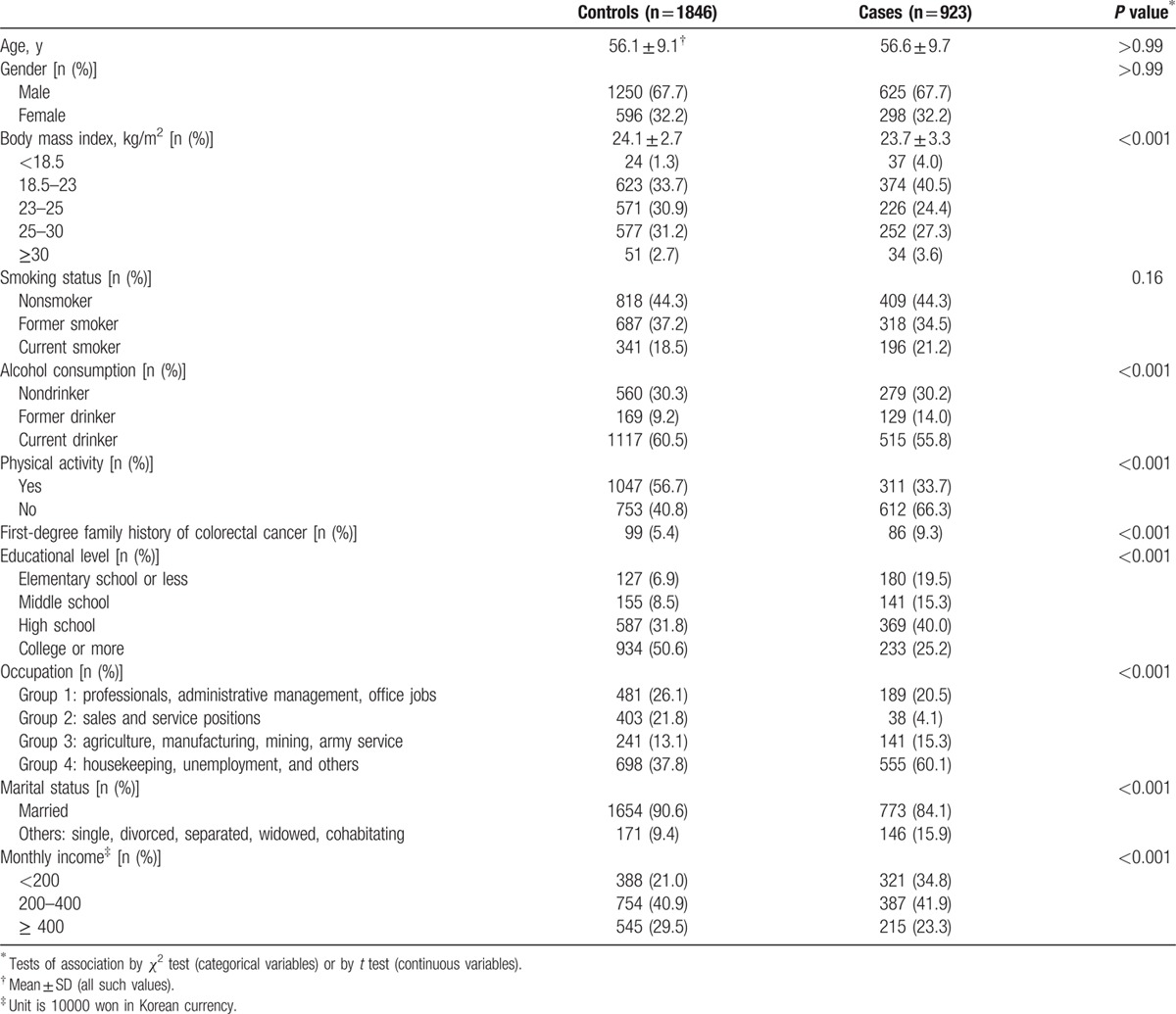
General characteristics of the study subjects (n = 2769).

The 3 major dietary patterns derived by using exploratory factor analysis and the factor loading matrices for both genders are shown in Table 2. Factors were interpreted as dietary patterns and named based on the food groups with high factor loadings. Pattern 1 was termed the traditional pattern because it showed high loadings of traditional food items regularly consumed in the Korean population, including vegetables, tubers, seaweeds, fish, soy, mushrooms, and seasonings. Pattern 2 was termed the Westernized pattern due to high factor loadings for a variety of different meats (red meat, meat by-products, and poultry), fast foods rich in carbohydrates (cakes, pizza, bread, hamburger, and noodles), oil and sugar.^[35–37]^ Pattern 3 was termed the prudent pattern, which included high loadings of fruits, milk and dairy products, cereals, nuts, and a low intake of refined grains and kimchi. The proportion of total variance explained was 24.2% for men (11.1%, 7.5%, and 5.6% for patterns 1, 2, and 3, respectively) and 25.3% for women (10.1%, 9.1%, and 6.1% for patterns 1, 2, and 3, respectively).

**Table 2:**
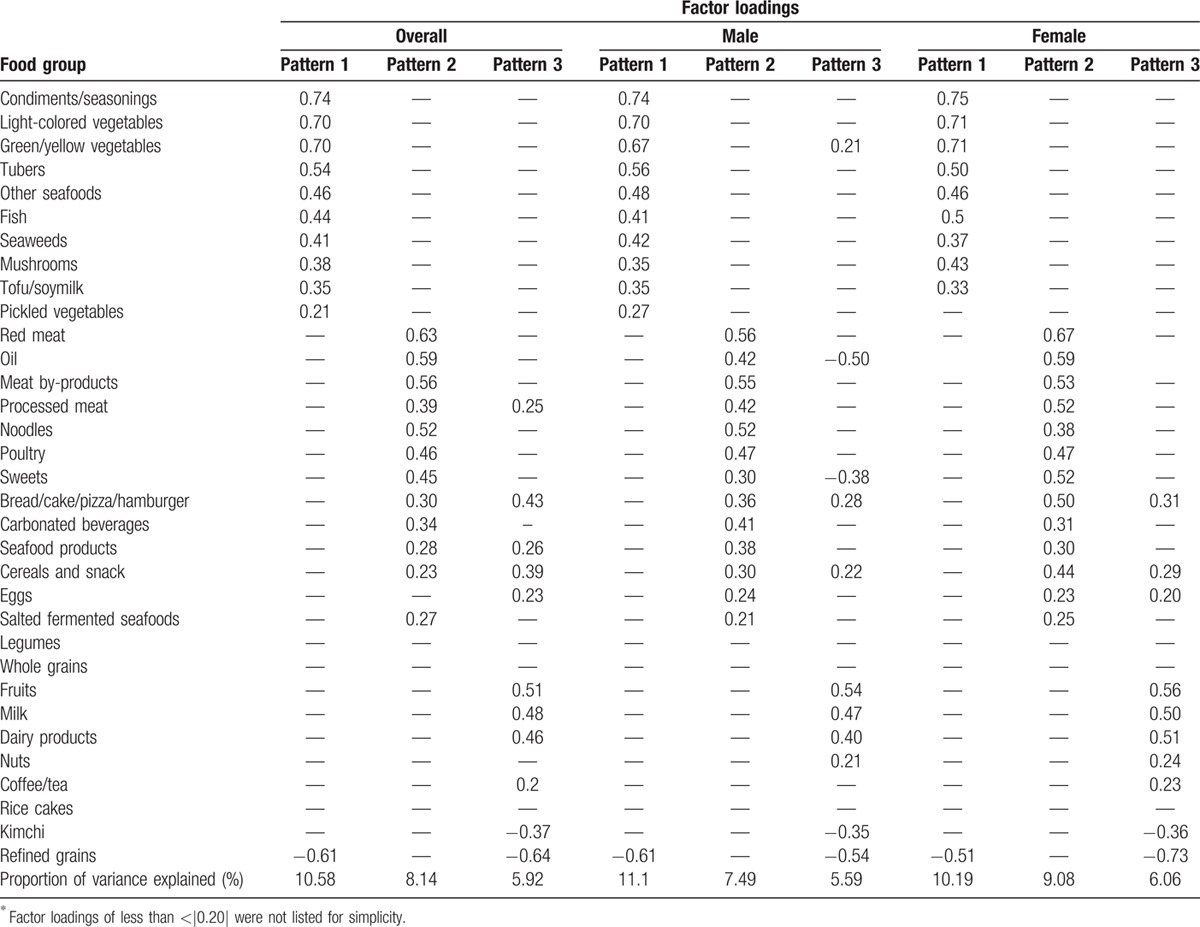
Factor loading matrix for the 3 major patterns identified by factor analysis.^∗^

Based on the intake tertiles of pattern score, the ORs (95% CIs) were obtained to explain CRC risk association with each dietary pattern in both genders adjusting for potential confounders (Table 3). Comparing the highest tertile to the lowest, a higher risk of CRC was associated with the Westernized pattern [OR = 2.35 (1.78–3.09)], and a reduced risk of CRC was related to both the traditional pattern [OR = 0.35 (0.27–0.46)] and the prudent pattern [OR = 0.37 (0.28–0.48)]. Polytomous logistic regression models were fitted to clarify the effects of the defined dietary patterns in association with different CRC subsites. In males (Table 4), a reduced risk of rectal cancer was significantly associated with the traditional pattern [OR = 0.45 (0.30–0.67)]. In females (Table 5), a reduced risk was also significantly associated with the traditional pattern [OR= 0.31 (0.12–0.78) for proximal colon cancer; OR = 0.53 (0.29–0.97) for distal colon cancer; OR = 0.31 (0.16–0.59) for rectal cancer]. For rectal cancer, a higher risk was significantly associated with the Westernized pattern [OR = 3.02 (1.60–5.72)] among females. In both genders (Table 3), the prudent pattern was inversely related to the risk of CRC at all cancer subsites [OR = 0.32 (0.19–0.52) for proximal colon cancer; OR= 0.37 (0.25–0.55) for distal colon cancer; OR = 0.38 (0.27–0.54) for rectal cancer].

**Table 3:**
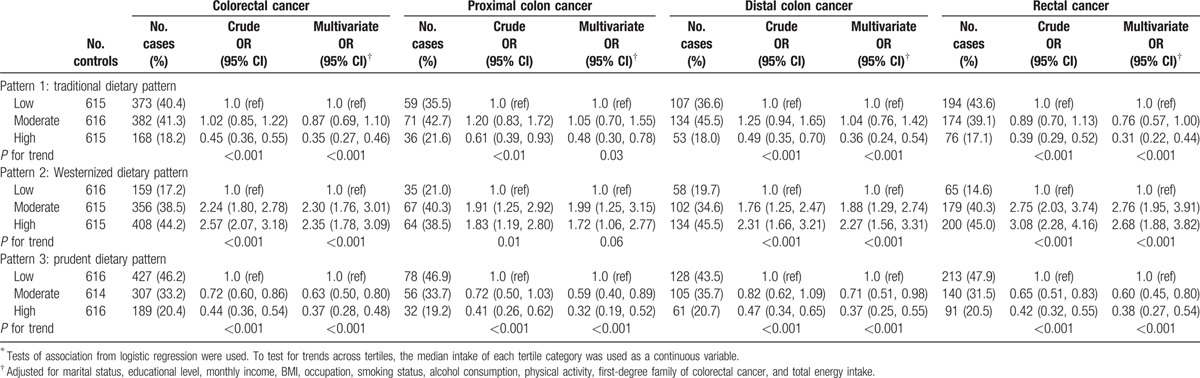
Colorectal cancer risks by anatomical locations according to dietary intake of the identified dietary patterns^∗^ for both genders.

**Table 4:**
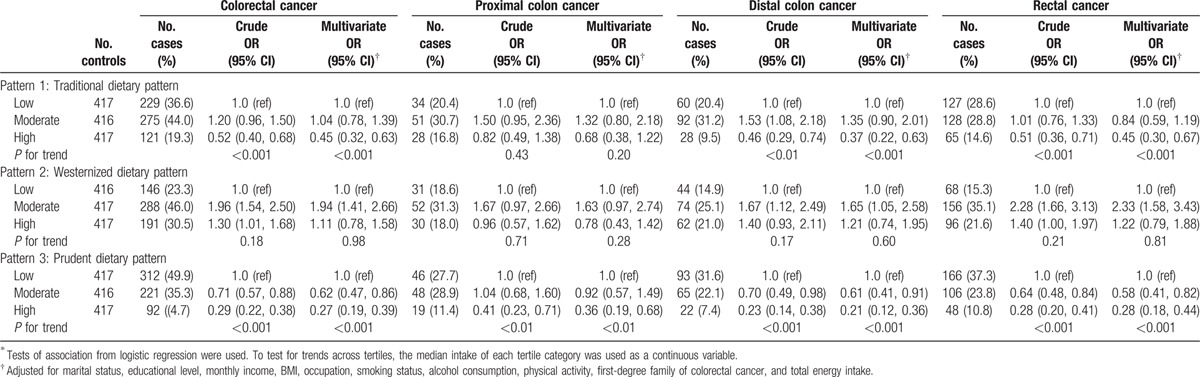
Colorectal cancer risks by anatomical locations according to dietary intake of the identified dietary patterns^∗^ for men.

**Table 5:**
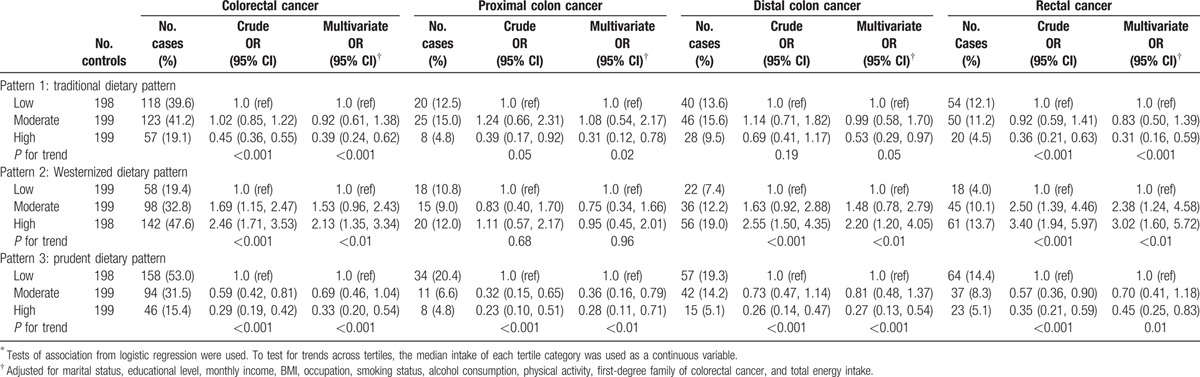
Colorectal cancer risks by anatomical locations according to dietary intake of the identified dietary patterns^∗^ for women.

## 4 Discussion

Overall, the findings of the current study demonstrate a significant positive association between the Westernized diet and the risk of CRC. Significant reductions in CRC risk were observed with the traditional and prudent dietary patterns, indicating that these patterns confer a protective effect against CRC. In particular, the prudent pattern was associated with a significantly lower cancer risk across all CRC subsites in both genders. Based on the dietary cultures or customs of different populations, dietary patterns might vary at local or national scales. Furthermore, food consumption patterns can change over time depending on food availability and preferences.^[20]^ Due to the subjective decision-making criteria used when performing factor analysis, the characteristics of a particular dietary pattern might not be consistent with those observed in other studies conducted in different populations.^[38,39]^ Moreover, the same dietary pattern term might differ slightly between Western and Asian populations based on food composition, which may lead to difficulties with reproducibility across different populations.^[23]^ In this study, the traditional dietary pattern was generally characterized by a higher intake of healthy foods of plant or animal origin, such as vegetables, tubers, fish, seaweeds, mushrooms, and soybeans. The Westernized dietary pattern was defined according to previous studies that have examined the Western-style diet, which includes high proportions of meat and processed meat, meat by-products, fast foods, and sweets.^[40,41]^ The prudent dietary pattern was defined by a diet low in refined grains and kimchi and high in fruits, milk and dairy products, and nuts.

Considering the increased risk of CRC, the Westernized dietary pattern, which involved higher consumption of meats, oil, carbohydrates, and sugar than did the other 2 patterns, showed a positive association with CRC risk,^[35–37]^ as we had hypothesized. The representative food components of this pattern were found to be disadvantageous in terms of CRC and have been investigated for their carcinogenic effects in numerous epidemiological studies. A case-control study conducted in Korea that involved a food group-based analysis showed that high red meat intake increased the odds of CRC,^[42]^ which was consistent with previous studies.^[10,43,44]^ There is supporting evidence of a dose–response relationship between red and processed meat consumption and the development of CRC; it was reported in a meta-analysis including 10 cohort studies that the CRC risk increased by 17% when an average of 100 g red meat had been consumed daily, and by 18% for a average of 50 g/day processed meat consumption (e.g. eating 2 slices of bacon a day).^[17,18,45]^ In addition, it has been suggested that the chemicals produced during the cooking process and culinary usage are more likely to increase the CRC risk.^[46]^ Studies on CRC have also revealed significant positive associations between CRC and exposure to dietary heterocyclic amines.^[47,48]^ Several studies conducted in industrialized countries indicate that Western diets rich in red and processed meat, refined starches, sugar, and saturated and trans-fatty acids are closely associated with an increased risk of CRC.^[21,30,46,47]^ A systematic review of diet and CRC risk in Asia reported that red and processed meats, animal fats, cholesterol, high-sugar foods, and refined carbohydrates were positively associated with CRC risk.^[48]^ Moreover, previous studies have suggested that modifications of the Western-style diet could substantially reduce the incidence and mortality of CRC by reducing the consumption of red meat and increasing consumption of foods of plant origin.^[39,49]^

With respect to modifying the Western diet, 2 dietary patterns can be recommended to reduce the risk of CRC in the Korean population. First, our findings indicated that a higher consumption of the traditional pattern significantly reduced the risk of CRC, particularly the risk of rectal cancer in both genders. In addition, a significant association between the prudent pattern and a decreased risk of CRC at all subsites was observed in both genders. Previous studies conducted in diverse populations have suggested that a diet consisting of a high intake of vegetables, fruits, and cereals might be protective against CRC.^[50,51]^ Studies conducted in the United States also support that dietary patterns characterized by a low frequency of meat and fat-rich foods and frequent consumption of fruit and vegetables are associated with a reduced risk of CRC,^[22]^ particularly colon cancer.^[52]^ Among Caucasian participants in the North Carolina Colon Cancer Study, a dietary pattern rich in fruits and whole grains was associated with a reduced risk of rectal cancer.^[53]^ Vegetables and fruits are rich in fiber, antioxidant vitamins, carotenoids, folic acid, and other phytochemical compounds, which might have preventive effects against colorectal carcinogenesis.^[54]^ The inclusion of plant-based foods with a high anticancer phytochemical content has been reported to be beneficial in terms of CRC prevention.^[49,55]^ Studies reporting that highly refined cereals might increase the risk of cancer also support the protective effect of the prudent pattern, which includes a low intake of refined grains. A high rate of digestion of refined grains and the consequent increases in plasma insulin and insulin-like growth factor 1 have been related to an increased risk of CRC.^[56,57]^ Furthermore, a meta-analysis indicated that a high intake of milk with a relative risk (RR) of 0.83 (95% CI = 0.74–0.93) and a high intake of total dairy products with a RR of 0.81 (95% CI = 0.74–0.90) were associated with reductions in CRC risk compared with low intake of these foods.^[12]^

The strengths of the present study include a methodological approach that employed principal component analysis to consider the complexity and interactions within or among the dietary patterns of individuals in a specified population.^[16,19]^ This approach accounted for the cultural diversities in each population with respect to different dietary patterns and habits. Identification of the major food groups contributing to each pattern allowed us to suggest dietary modifications that could reduce cancer risk in the Korean population. Another strength of the study was the relatively large number of included cases compared with other previous case-control studies of diet and CRC risk conducted in Korea.^[42,58]^ An additional advantage of this study was the analysis of the risk association between each dietary pattern and distinct CRC location for each gender. Because dietary etiological factors may vary among sites, such anatomical stratification may help in further understanding cancer risk and prevention. Some previous studies from the United States conducted analyses based on particular anatomical subsites to assess the association with dietary patterns. Although dietary patterns had different effects on the risk of colon cancer depending on anatomical subsite,^[50,59]^ the reasons for these differences remain unclear, as described in a systematic review.^[30]^

This study has some potential limitations. First, recall of dietary habits may differ between men and women, or between cases and controls due to different levels of dietary knowledge and health compliance. Furthermore, case-control studies are prone to recall bias, and cancer patients are more likely to recall perceived unhealthy dietary habits compared with healthy controls.^[49]^ Second, the use of factor analysis to derive dietary patterns involves subjective decisions when consolidating food items into food groups (variables), extracting the number of factors, and labeling of the patterns.^[19,38]^ Last, the case and control groups were recruited from the same hospital of the NCC in Korea. However, this does not ensure that 2 groups were from the same source population due to the location and specialization of the medical facility.

In conclusion, the Westernized dietary pattern was associated with an elevated risk of CRC, whereas the traditional and prudent patterns were associated with a decreased risk of rectal cancer and all types of CRC, respectively. Our findings suggest that individuals who have a high intake of meat and sugar should be made aware of their increased risk for CRC and of the preventive strategies. To prevent CRC, transitioning from a Westernized dietary pattern to a more traditional pattern is recommended; this can be accomplished by consuming more foods of plant or natural origin in combination with regular intake of fruits, milk, and dairy products, which are major contributors to the prudent dietary pattern. The dietary recommendations described in this study can be used to support guidelines for CRC prevention and to develop public health policies.

## Supplementary Material

Supplemental Digital Content
