# Supplementary material for: Dietary patterns and colorectal cancer risk in a Korean population: A case-control study
Source: Medicine (Baltimore). 2016 Jun 24;95(25):e3759. doi: 10.1097/MD.0000000000003759 (PMC4998300; doi:10.1097/MD.0000000000003759)
Supplement: Supplemental Digital Content [file medi-95-e3759-s001.doc]

| Appendix. Food groups and their contents | |  |
| --- | --- | --- |
| Food groups | Contents | |
| Whole grains | Glutinous rice, brown rice, barley, black rice, foxtail millet, sorghum | |
| Refined grains | Wheat flour, white rice | |
| Noodles | Ramyon (instant noodle), Chinese noodle, udong, wheat noodle | |
| Bread/cake/pizza/hamburger | Bread, pizza, hamburger, sandwich, cake, pie, doughnut | |
| Cereals and snack | Cereals, rice crispy, corn flakes, snack, biscuit, cookies, cracker | |
| Rice cakes | Rice cakes | |
| Tubers | Potatoes, sweet potatoes, potato starch, cellophane noodles | |
| Sweets | Honey, sugar, starch syrup, candies, caramel, chocolates, fruit jams | |
| Legumes | Soybeans, green peas, black beans | |
| Tofu/soymilk | Tofu, soft tofu, fried tofu, soybean curd residue, soy milk | |
| Nuts | Peanuts, almonds, pine nut, sesames | |
| Green/yellow vegetables | Green chilli, red chilli, red-pepper leaf, leaf beet, carrots, spinach, lettuce, leeks, broccoli, tomatoes, tomato juice, tomato paste, green onion, pumpkin, zucchini, korean lettuce, squash | |
| Light-colored vegetables | Garlic, radishes, ginger, celery, brussels sprouts, onion, cucumber, bean sprouts | |
| Pickled vegetables | Pickled radishes, pickled cucumber, pickled garlic | |
| Kimchi | Korean cabbage, Korean cabbage (non-red pepper), seasoned cubed radish roots, na-bak, dong-chi-mi | |
| Mushrooms | Oyster mushroom, matsutake mushroom, button mushroom, oak mushroom, winter fungus | |
| Fruits | Strawberries, oriental melon, melon, watermelon, peaches, plum, bananas, persommons, tangerine, pears, apples, oranges, grapes, fruit juices, canned fruits | |
| Red meat | Beef (loin, tender loin, ribs), pork (loin, tender loin, shoulder. ribs, belly) | |
| Meat by-products | Beef (small intestine), pork (feet) | |
| Processed meat | Ham, bacon, sausages | |
| Poultry | Chicken | |
| Eggs | Eggs, quail's eggs | |
| Fish | Fatty fish (mackerel, pacific saury, spanish macherel, tuna), lean fish (scabbard fish, bastard halibut, Alaska pollack, yellow corvina, bone fish (anchovy) | |
| Salted fermented seafoods | Salted fish, salted clams, pickled shrimps, fermented squid | |
| Seafood products | Fish pastes | |
| Other seafoods | Clam, oyster, cockle, mussel, conch, crab, shrimp, small octopus, squid | |
| Seaweeds | Laver, sea tangle, sea mustard | |
| Milk | Whole milk, low-fat milk, skim milk | |
| Dairy products | Liquid yogurt, curd yogurt, cheese, ice cream, sherbet | |
| Oil | Butter, margarine, sesame oil, soybean oil, coffee cream | |
| Carbonated beverages | Soda | |
| Coffee/tea | Instant coffee, green tea, citron tea, ginseng tea, sweet rice drink | |
| Condiments/seasonings | Soy sauce, mustard, red pepper powder, red pepper paste, sesame salt, soybean paste, salt, vinegar, tomato ketchup, pepper powder | |
